# 2403. Outcomes in Infective Endocarditis due to *Granulicatella* species and *Abiotrophia defectiva*

**DOI:** 10.1093/ofid/ofad500.2023

**Published:** 2023-11-27

**Authors:** Cindy McCartney, Patricia Bartley, Nabin K Shrestha

**Affiliations:** Cleveland Clinic Foundation, Cleveland, Ohio; Cleveland Clinic, Cleveland, OH; Cleveland Clinic Foundation, Cleveland, Ohio

## Abstract

**Background:**

Infective Endocarditis (IE) due to *Granulicatella sp.* and *Abiotrophia defectiva* (formerly known as nutritionally variant streptococci - NVS) remains a clinically important entity given difficulty in diagnosis, optimal management, and possible treatment failure outcomes. These microorganisms account for roughly 4-8% of all IE cases. To the best of our knowledge this study entails the largest single center registry in the US of *Granulicatella sp.* and *A. defectiva* IE looking at patient characteristics, survival and relapse outcomes and susceptibility data.

**Methods:**

Retrospective chart review of 35 patients (13 *A. defectiva*, 20 *Granulicatella sp.* and 2 labelled as NVS) with IE admitted to our facility from January 1^st^, 2008, to January 1^st^, 2023.

**Results:**

Patient characteristics including valves affected, presenting symptoms and complications can be seen in Table 1. Seven patients (20% - 5 for *A. defectiva* and 2 for *Granulicatella sp.*) had failed medical management at outside facilities prior to admission at our center. Valve replacement or repair was performed on 31 (88.6%) patients. Molecular testing was performed on 28 (80%) isolates of which all had positive identification. Susceptibilities were obtained in 22 (63%) isolates as summarized in Table 2 below. All isolates were found to be vancomycin-susceptible, and varying degrees of susceptibilities to ceftriaxone and penicillin depending on the species. Vancomycin therapy was used in 11 (31.4%) patients, ceftriaxone in 19 (54.3%) patients (combination of vancomycin and ceftriaxone in 3 patients), and penicillin in 8 (22.9%) patients. Adjuvant aminoglycoside therapy was used in only 2 (5.7%) patients (one combination with ampicillin and one with vancomycin). Average length of IV antibiotic therapy was 6 weeks post-surgery, and 5 patients were treated with an oral tail after IV antibiotics. Survival curves since hospitalization are demonstrated in Figure 1. There were no treatment failures or relapses of IE due to these organisms.

**Figure:**
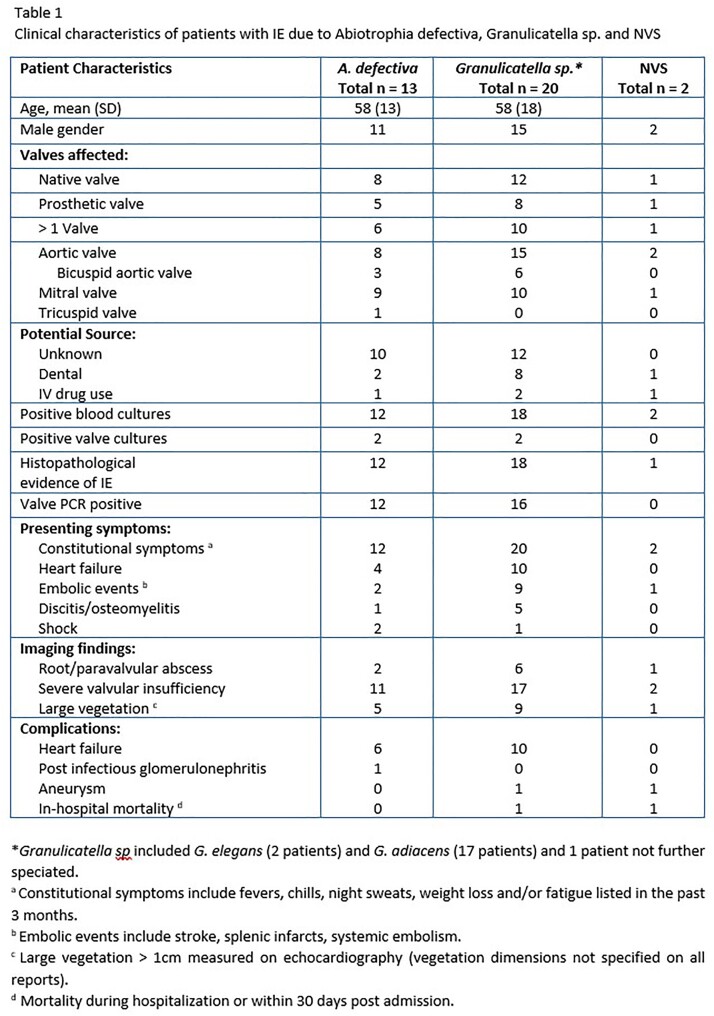

**Figure:**
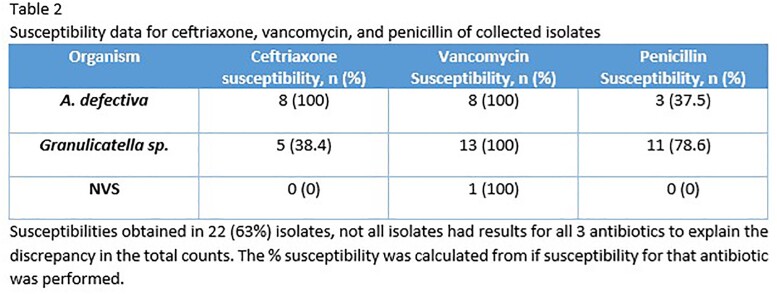

**Figure:**
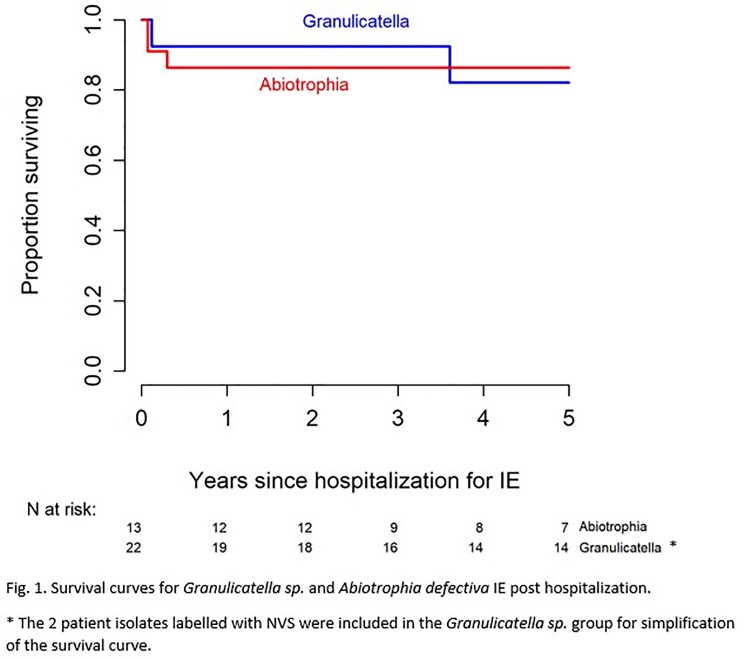

**Conclusion:**

The majority of patients presented with constitutional symptoms, heart failure and embolic events. Five-year survival exceeded 80% and there were no relapses in this series. Most importantly, outcomes were excellent without the use of aminoglycosides contrary to guideline recommendations.

**Disclosures:**

**All Authors**: No reported disclosures

